# The ABCs of wheeze: Asthma and bacterial communities

**DOI:** 10.1371/journal.ppat.1007645

**Published:** 2019-04-25

**Authors:** Naomi G. Wilson, Ariel Hernandez-Leyva, Andrew L. Kau

**Affiliations:** Department of Medicine and Center for Women’s Infectious Disease Research, Washington University School of Medicine, St. Louis, Missouri, United States of America; Nanyang Technological University, SINGAPORE

## Is asthma a problem of microbial ecology?

Asthma is a common respiratory illness affecting approximately 8% of Americans and is characterized by symptoms of wheeze, cough, and shortness of breath. These symptoms are caused by an inappropriate sensitization to an environmental antigen, or allergen, that leads to airway inflammation upon reexposure. These responses are typically mediated by allergic T cells (T-helper 2 [Th2]), which trigger eosinophilic inflammation characteristic of allergic asthma. Multiple factors contribute to the clinical risk for asthma. A genetic predisposition to allergy and asthma, called atopy, is a well-established feature in its development. Additionally, environmental factors including exposure to allergens, birth delivery mode, diet, and childhood surroundings may also modify asthma risk.

More recently, the role played by the body’s endogenous microbial communities (microbiota) has emerged as a new component to this puzzle. Healthy environmental microbial exposures not only help the neonatal immune system, including humoral and innate components, to mature into an adult-like state after birth but also likely influence immune function throughout life [1]. Maladaptive microbiota–host interactions have likewise been linked to the development of disease. A number of studies across a wide variety of illnesses have changed the way that we regard “pathologic” microbe–host interactions to include the concept of “dysbiosis,” or dysfunctional microbial communities that contribute to pathology. These dysbiotic communities fail to adequately perform the normal functions of a healthy microbiota in educating the immune system, processing dietary components, producing bioactive compounds, and more, which can profoundly impact host health and disease susceptibility. Dysbiotic gut and airway microbial communities have been implicated in asthma pathogenesis, indicating that a comprehensive understanding of the disease will require a detailed understanding of resident bacterial–host interactions.

## How could microbial community dynamics promote allergy?

Our microbiota can shape immune responses in numerous ways, including interacting directly with host immune components, altering metabolic functions, or preventing or promoting pathogen invasion. Understanding microbial ecology in the context of the host environment is therefore an increasingly important objective as we seek to understand how microbes may facilitate allergic inflammation. Even in a stable, nondiseased state, the mucosal environment in which most commensal microbes reside presents a highly dynamic and complex habitat with distinct niches and available resources. Further complicating this interaction, microbes in the mammalian host have the capacity to alter their environment through their interaction with the host immune system. These microbe-stimulated changes to the environment may be an opportunity for bacteria to alter their habitat in their favor, either producing a unique niche or creating a barrier to competing microbes. *Bacteroides fragilis*, for example, has recently been reported to take advantage of the host intestinal immunoglobulin A (IgA) response to generate a unique niche that enables resistance to displacement by other microbes [2]. Although beneficial to a specific microbe, such immunomodulation may have consequences for the host.

**Fig 1:**
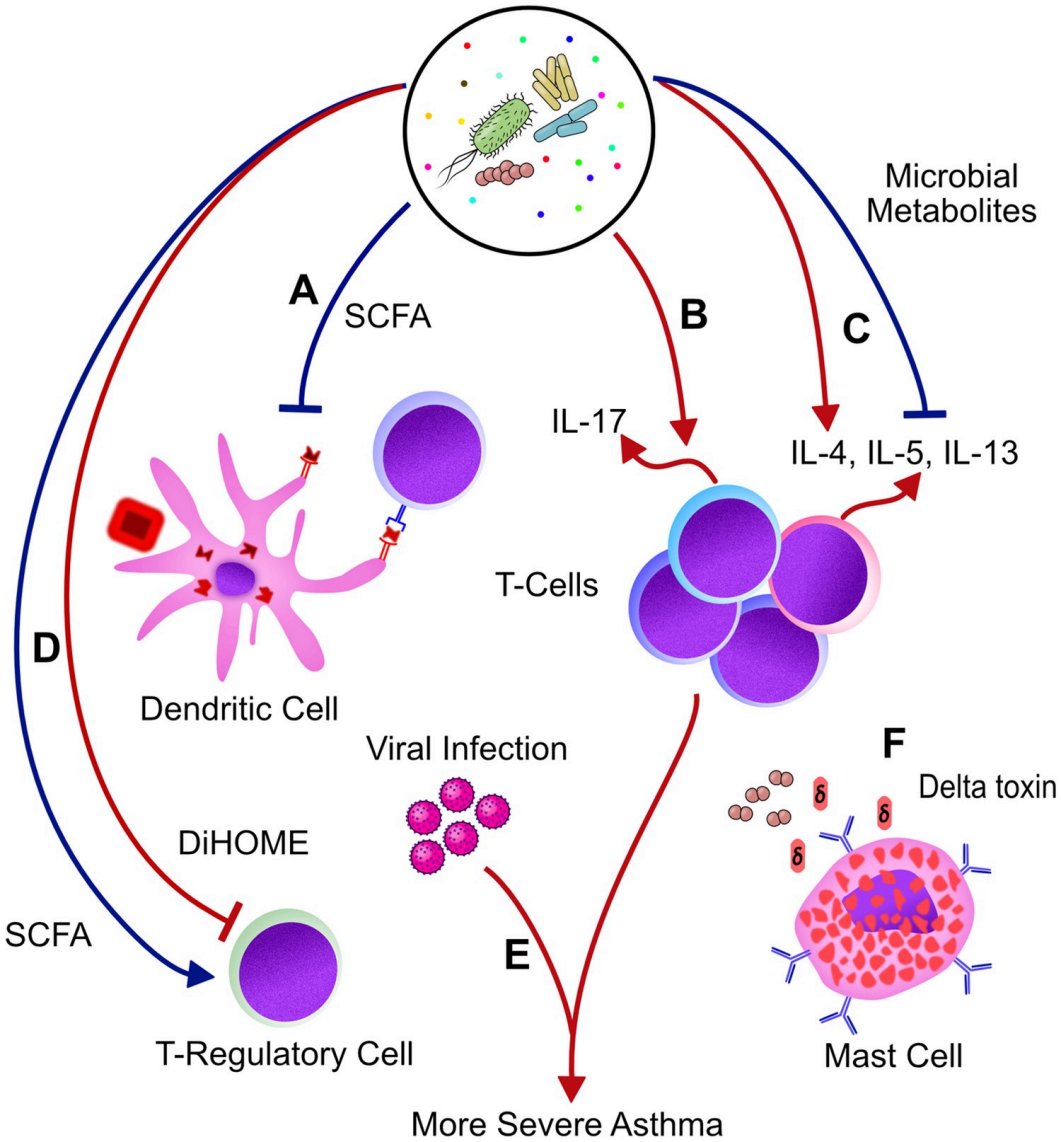
Bacterial communities and metabolites impact multiple stages in the well-established pathway of allergic inflammation, both offering potentially protective effects (blue arrows) and exacerbating allergic inflammation (red arrows). (A) SCFAs produced by gut microbiota enhance bone marrow production of dendritic cells and macrophages with increased phagocytic capacity but reduced ability to stimulate Th2 responses in the lung [33]. (B) Bacteria found in the airway have been associated with increased markers of Th17 inflammation [20,21,24]. (C) Microbially derived metabolites may directly stimulate or inhibit [35] Th2 development [31]. (D) Microbially derived SCFAs found in the healthy gut promote Treg differentiation [34], whereas metabolites like DiHOME produced by asthmatic gut microbiota inhibit Treg proliferation [31]. (E) Viral respiratory tract infections are associated with increased risk for asthma and exacerbations [8]. (F) *S*. *aureus* colonizes the skin of AD patients depleted of antimicrobial peptides by allergic inflammation and carries virulence factors that influence the host immune system to sustain the inflamed state. *S*. *aureus* delta toxin, for example, acts on pathways not associated with immunoglobulin E to stimulate mast-cell degranulation near the site of colonization [3]. Investigating the precise mechanisms by which bacteria can worsen or mitigate asthma and allergic inflammation will offer novel probiotic candidates and biomarkers with therapeutic potential. AD, atopic dermatitis; DiHOME, 12,13-dihydroxy-9Z-octadecenoic acid; IL, interleukin; SCFA, short-chain fatty acid; Th2, T-helper 2; Th17, T-helper 17; Treg, T regulatory cell.

Like asthma, atopic dermatitis (AD), also called eczema, is a chronic inflammatory skin disease triggered by Th2 allergic inflammation and is characterized by itchy, dry, and red skin rashes. Interestingly, AD is associated with a defined skin microbial signature, dominated by *Staphylococcus aureus*, that likely plays a role in AD manifestations. *S*. *aureus* colonization in AD has been associated with disease severity [3,4] and may directly promote AD by activating mast cells through the production of δ-toxin [5] and contributing to Th2 activation [6] (Fig 1F). Inflamed skin, in turn, down-regulates the expression of antimicrobial peptides [7], which permits further proliferation and persistence of *S*. *aureus*, potentially amplifying the course of disease. Although it is not yet clear what the specific role of such interactions is in allergic responses, these examples underscore how the factors that govern the microbial ecology of our microbiota may contribute to asthma and emphasize the potential importance of microbial communities residing in close association with allergic inflammation in the airway.

## Is microbial community composition in the upper airway a risk factor for asthma?

Early-childhood viral respiratory tract infections have long been recognized to increase the risk of asthma and contribute to asthma exacerbations [8] (Fig 1E). Bacterial infections have likewise been implicated in promoting asthma exacerbations, but the roles of airway-colonizing bacteria in shaping allergic airway responses are only recently being explored. Similar to the gastrointestinal tract [9], the anatomy of the airway plays an important role in shaping microbial communities along the respiratory tract. The nasal vestibule is perpetually exposed to the environment and encounters a constant barrage of debris, microbes, and microbial products, which presumably contribute to the relatively high density of microbes in the upper airway. Beyond the nasal vault, air, remaining particles, and microbes aspirated from the oropharynx can pass into the trachea, which is often defined as the beginning of the lower respiratory tract. Particles and microbes that are deposited in the lower airway are entrapped in mucus and returned back up to the esophagus by mucociliary clearance. These natural clearance and relocation mechanisms contribute to shared members but marked differences in abundance between the upper- and lower-airway bacterial communities [10].

In the upper respiratory tract, specific bacterial taxa have been associated with the development of asthma. Multiple studies have reported that neonates colonized with *Streptococcus pneumoniae*, *Haemophilus influenzae*, or *Moraxella catarrhalis* are at an increased risk for later diagnosis of asthma compared with uncolonized children [11,12]. The presence of these asthma-associated microbes in infancy does not reflect mere chance exposure, because their appearance is associated with acute viral respiratory infections as well as multiple other environmental factors including antibiotic usage and daycare attendance [13]. These findings have been extended to early childhood through 16S rRNA sequencing of nasopharyngeal aspirates from a cohort of children carefully monitored for respiratory infections during the first 5 years of life. Confirming earlier reports, these studies found that the same three taxa were predictors of future diagnosis of asthma [14]. Additionally, the high temporal resolution of sampling enabled the observation that illness-associated taxa bloom in abundance preceding viral infection. Perhaps most intriguingly, this study found a time period during early childhood in which colonization with *Streptococcus*, *Moraxella*, and *Haemophilus* in the upper airway predicted later wheeze (a precursor to asthma) in children [14]. A similar “critical window” of microbial exposure important for later asthma development has also been described in the gut microbiota and could be scientifically and clinically valuable because it marks a time frame for potential intervention.

## Is there a lung microbiota, and does it play a role in asthma?

The lung itself was regarded as a sterile environment in healthy individuals, maintained by intrinsic clearance mechanisms, until culture-independent techniques revealed the presence of a lung microbiota, though at a markedly decreased abundance compared with the upper airway [15]. Although studying microbial communities in the lungs is technically challenging because of low bacterial biomass and the difficulty in acquiring samples [16], differences in the lung microbiota are associated with asthma [17] and other pulmonary diseases [18]. Airway microbes may also exert an important effect on asthma even after it is established by modulating response to therapy [19] or altering the character of inflammatory response [20,21]. Moreover, specific alterations to the lung microbiota have been associated with distinct asthma phenotypes. For instance, Proteobacteria from lower-airway samples have been associated with neutrophilic asthma, an endotype of asthma associated with T-helper 17 (Th17) responses [20,21] (Fig 1B). This ability of airway microbes to alter clinical features of asthma parallels our evolving understanding of asthma as a highly heterogeneous condition, with many unique pathophysiologies (referred to as endotypes) that all ultimately lead to asthma’s characteristic features: airway hyperresponsiveness and obstruction. Whereas the endotype associated with “classical” allergic asthma is mediated by Th2 inflammation, multiple endotypes have been described [22].

Although there are multiple clinical studies validating the association between upper- and lower-airway microbes and asthma, further efforts will be needed to unravel how these bacteria modify allergic responses. The ability of upper-airway microbes to shape allergic responses has been investigated in animal models. Inoculation of neonatal mice with nontypeable *Haemophilus* results in transient infection and later susceptibility to worsened allergic airway inflammation [23]. Exposure to *M*. *catarrhalis* has likewise been found to intensify allergic inflammation in mice through the induction of interleukin 17 (IL-17), resulting in more recruitment of neutrophils and eosinophils to the lungs [24]. Using these animal models, we have additionally gleaned that early colonization of the airway by bacteria helps shield mice from asthma through programmed death ligand 1 (PD-L1)-dependent induction of T regulatory (Treg) cells [25]. Microbial colonization in the first 2 weeks of life induces PD-L1 expression on dendritic cells, which signal Tregs to persist in the immune system. The specific host–bacterial interactions that promote allergic airway inflammation remain enigmatic, however, and require additional investigation.

## How do gut microbes shape allergic inflammation in the lung?

The vast community of microbes inhabiting the gut has been documented to influence a surprisingly diverse set of diseases, even illnesses that typically involve pathologies at anatomically distant sites, like multiple sclerosis [26,27] and arthritis [28]. The influence of gut microbes on pulmonary diseases has been referred to as the “gut–lung axis,” and several studies have implicated intestinal microbial communities in the pathogenesis of asthma, particularly in early childhood. Although multiple pathways probably underlie the communication between gut microbial communities and the lung in asthma [29,30], microbiota-derived metabolites are emerging as a particularly compelling example.

For instance, the composition of neonatal gut microbial communities can acquire configurations with decreased representation of *Akkermansia*, *Bifidobacterium*, and *Faecalibacterium*, which predict later development of allergy and asthma [31]. Coculture of sterile fecal extracts generated from these dysfunctional microbial communities with human lymphocytes promotes the development of allergy-inducing Th2 cells while inhibiting the differentiation of Treg cells, directly implicating microbially derived products in conferring susceptibility to allergic disease (Fig 1C and 1D).

Furthermore, investigators studying the Canadian Healthy Infant Longitudinal Development cohort identified four taxa in neonates—*Faecalibacterium*, *Lachnospira*, *Veillonella*, and *Rothia* (FLVR)—that corresponded to later protection from asthma. When these four bacteria were supplemented to an asthmatic gut microbial community in gnotobiotic mice, animals receiving FLVR taxa experienced protection from allergic airway inflammation compared with controls that received no supplementation. Colonization with FLVR also corresponded to increases in the amounts of fecal acetate [32], a short-chain fatty acid (SCFA) known to protect from asthma [33]. Differences in SCFAs have been observed in 3-month-olds who displayed atopy and wheeze at 1 year [32]—symptoms that are predictive of later asthma. Moreover, SCFAs are known to influence Treg differentiation and function (e.g., [34]), which may contribute to asthma. Additionally, SCFAs generated by the gut microbiota have also been demonstrated to alter dendritic-cell recruitment and function within the lungs and reduce Th2 inflammation [33,35] (Fig 1A). Intriguingly, FLVR were protective only when present during the first 100 days of life, which suggests a critical window of gut colonization during which exposure to key organisms can prevent later disease. This phenomenon is similar to the critical window observed for airway colonization [14], suggesting that exposure to particular microbial communities during a crucial moment in early life results in a lasting restructuring of the host’s immune system.

## Concluding remarks

Our growing appreciation for the role of commensal microbes in human health has placed the microbiota among the important factors contributing to asthma pathogenesis. Although the mechanisms by which endogenous bacterial communities affect asthma are still being elucidated, the enticing potential for therapeutic interventions will ensure continued interest and exploration on the influence of microbial communities on allergic diseases. Perhaps most compellingly, unlike host genetics and environmental exposures, which are known risk factors for allergic diseases, the microbiota presents a more easily modifiable feature in asthma development. One potential approach is to design probiotics that alter the composition or function of respiratory or gastrointestinal microbial communities in at-risk children. The promise of this approach relies on identifying individuals who would benefit from a probiotic intervention and exploiting the “critical window” in early life, during which microbial exposures can shape later asthma [14,31,32]. However, before we can benefit from microbiota-directed therapeutics in asthma, it is necessary to further define the mechanism(s) by which gut and airway microbes protect from the disease and to assist the identification of therapeutic candidates and biomarkers. Together, these new insights signify the beginning of a novel paradigm to understand the etiology of asthma as an emergent condition resulting from the codevelopment of microbial communities and host immunity. Innovations from this research may lead to transformative advances in our understanding and treatment of asthma.
